# A Tailored Home-Based Training Program Improved Ataxia Severity and Participation in Adults With ARSACS

**DOI:** 10.1007/s12311-025-01816-z

**Published:** 2025-03-17

**Authors:** Isabelle Lessard, Cynthia Gagnon, Marjolaine Tremblay, Laura Girard-Côté, Isabelle Côté, Mylène Aubertin-Leheudre, Elise Duchesne

**Affiliations:** 1 Groupe de recherche interdisciplinaire sur les maladies neuromusculaires (GRIMN), Centre intégré universitaire de santé et de services sociaux du Saguenay–Lac-St-Jean, Québec, Canada; 2 Centre ÉCOBES-Recherche et Transfert, Cégep de Jonquière, Québec, Canada; 3 Faculté de médecine et des sciences de la santé, Université de Sherbrooke, Québec, Canada; 4 Centre de recherche du Centre hospitalier universitaire de Sherbrooke (CRCHUS), Université de Sherbrooke, Québec, Canada; 5 École des sciences de la réadaptation, Faculté de Médecine, Université Laval, Québec, Canada; 6 Département des Sciences de l’activité physique, Faculté des sciences, Université du Québec à Montréal, Montréal, Canada; 7 Centre de recherche, Institut universitaire de gériatrie de Montréal (IUGM), CIUSSS du Centre- Sud-de-l’Île-de-Montréal, Montréal, Canada; 8 Centre interdisciplinaire de recherche en réadaptation et intégration sociale (CIRRIS), Institut de réadaptation en déficience physique de Québec, Québec, Canada; 9 CHU de Québec – Centre de recherche de l’Université Laval, Québec, Canada; 10 Faculté de Médecine - École des sciences de la réadaptation Pavillon Ferdinand-Vandry, 1050, avenue de la Médecine, Québec, G1V 0A6 Canada

**Keywords:** Spastic ataxia Charlevoix-Saguenay type, Exercise, Rehabilitation, Physical therapy modalities, Ataxia

## Abstract

Autosomal recessive spastic ataxia of Charlevoix-Saguenay (ARSACS) is a rare degenerative movement disorder impacting balance and mobility. Rehabilitation helps to reduce disease severity and increase the quality of life of people with ARSACS. However, rehabilitation programs require many trips to dedicated facilities, posing a significant challenge for individuals living with ARSACS. This study aimed to develop a home-based training program specific for adults with ARSACS and to document its effects on ataxia severity, balance, mobility, and participation. This non-blinded and non-randomised interventional study used a pre-post design with a control phase. The initial level of training difficulty was tailored to each participant using a standardized assessment tool. Participants trained three times a week for 12 weeks. Outcome measures included the BERG Balance Scale, Ottawa Sitting Scale, 10-meter Walk Test, 30-second Chair Stand Test, 10-Steps Test, LIFE-H, and Scale for the Assessment and Rating of Ataxia. The retrospective acceptability of the program was also assessed using Sekhon’s theoretical framework of acceptability. Fourteen participants (eight walkers, 50% men) completed the program (self-reported attendance rate rate: 75–100%) and seven dropped out. All outcome measures remained stable during the control phase. Ataxia severity and participation significantly improved after the 12-week home-based training program. At an individual level, clinical improvements in standing and sitting balance were noted for almost half of the participants, particularly for non-walkers. The eight participants who assisted to the focus group reported that the program was acceptable. This study indicates that tailored home-based training is safe, acceptable, and helps to reduce ataxia severity and participation restriction for adults with ARSACS.

## Introduction

Autosomal recessive spastic ataxia of Charlevoix-Saguenay (ARSACS) is one of the most prevalent autosomal recessive ataxias [1]. The incidence in the Charlevoix-Saguenay region (Province of Quebec, Canada) is one of the highest in the world, estimated at 1 in every 1 932 live-born infants, with a carrier rate of 1 in 22 inhabitants in the population [2]. This hereditary disease can be caused by a myriad of mutations in the *SACS* gene on chromosome 13q12, the c.8844delT being the most frequent [3–6].

ARSACS is characterized by neuropathic, pyramidal, and cerebellar impairments. There is a high level of variability among affected people in terms of clinical presentation, severity, and progression rate of impairments [7, 8]. ARSACS is slowly degenerative, with significant clinical changes observed over 2 to 4 years [9, 10]. ARSACS leads to several activity limitations, including major issues with mobility and balance [11]. For instance, wheelchair use on a daily basis begins around 40 years old, with high variability (17–58 years) [12]. Also, 37% of people with ARSACS who walk without a walking aid and 94% of those who use a walking aid are at high risk of falling when walking, performing transfers, or maintaining a standing position [11]. From the age of 20, people with ARSACS also reported an average of 18 falls per year compared to one fall per year for 20 to 30% of people aged 65 and over [13]. Walking speed is also decreased in the ARSACS population with many factors contributing to this decrease such as balance, lower limb motor control, and balance confidence [11]. All those motor impairments may drastically impact the quality of life by restricting their participation in daily and social activities [14, 15].

Only two exploratory studies were specifically carried out in the literature to investigate the effect of a tailored training program on people with ARSACS [16, 17]. Those studies have shown that physical training can improve balance and overall mobility limitations, which positively impacts the ability to perform activities of daily living. The first study conducted by Audet et al. [17] involved 12 participants in an eight-week training program that included physical activities, strength-power and aerobic training. They noted that participants gained strength and limb coordination, fell less often, and slightly increased their walking speed. In the second study by Lessard et al. [16], 10 participants were engaged in an eight-week rehabilitation program. In this study, participants significantly improved their balance, ataxia severity, and ability to perform activities of daily living. Both training programs required people with ARSACS to go to the gym or a dedicated rehabilitation center. However, adults with ARSACS could face significant barriers such as limited transportation access, as well as the costs and availability of these programs restricting the applicability of such programs.

This study aimed to (1) develop a safe and adapted 12-week home-based training program for adults with ARSACS; (2) document its effects on ataxia severity, balance, mobility and participation, and (3) evaluate its retrospective acceptability.

## Methods

This non-blinded and non-randomised interventional study used a pre-post design with a control phase. In the context of rare diseases with slow progression of deficits and limitations, this design allows for a larger number of participants in the intervention and reduces biases resulting from the marked heterogeneity within this population. To minimize assessment and intervention-related biases, two distinct physiotherapists were involved in the evaluations and interventions, ensuring the independence of these processes.

### Participants

Participants were recruited from the registry (*n* = 148) of the Saguenay neuromuscular clinic of the *Centre Intégré Universitaire de Santé et de Services Sociaux du Saguenay–Lac-St-Jean* (Quebec, Canada). A random sampling strategy stratified by sex and indoor mobility level (walker or non-walker) was used. Inclusion criteria were to have a genetic confirmation of the diagnosis, be between 18 and 50 years old, be able to perform a sit-to-stand transfer independently (with or without technical aids), have the medical authorization from the treating neurologist, speak English or French, and be able to give informed consent. People with ARSACS who were already meeting the physical activity recommendations (150 min/week), who already took part in a rehabilitation program, who live in a long-term care institution, or had other medical conditions leading to significant functional limitations or multiple morbidities preventing them from completing all assessments were excluded of this study. All participants meeting the criteria were contacted by phone for recruitment by a research professional.

### Ethical Considerations

The study was approved by the Ethics Review Board of the *Centre intégré universitaire de santé et de services sociaux du Saguenay–Lac-Saint-Jean* (#2021-047) and performed in accordance with the ethical standards as laid down in the 1964 Declaration of Helsinki and its later amendments. All participants provided written informed consent. The ClinicalTrials.gov Identifier was NCT05768750.

### Conception of the Standardized Assessment Tool and the Training Program

A training program named **P**romoting **A**utonomy by exer**C**is**E** (PACE)-ARSACS, was specifically developed for adults living with ARSACS by the research team, is available at https://www.grimn.ca/programme-pace-arscs. This team included a physiotherapist (PT) having clinical expertise with this population and an ARSACS patient partner [18]. The parameters of this training program are described according to the Modified Consensus on Exercise Reporting Template (CERT) for Therapeutic Exercise Interventions [19]. The exercises were developed by taking into account several criteria, including: (1) Stimulating the main impairments and activity limitations related to mobility among adults with ARSACS; (2) Applicability to all stages of mobility (walking without aid, walking with aid, using a wheelchair [independent in transfers]); (3) Progressiveness (sufficient number of difficulty levels allowing each exercise to progress over 12 weeks); (4) Maximum duration of 15 to 20 min; and (5) Feasibility (exercises that can be performed at home [safe, minimal equipment required, environmental considerations]). The exercises aimed to improve trunk control, balance in seated and standing positions, and facilitate the performance of sit-to-stand transfers. The selected exercises included in the training program were pretested by an ARSACS patient partner to evaluate the selection and feasibility of the exercises developed by the team, the progression order of the exercises based on the level of difficulty of their execution, and the clarity of the instructions for each exercise. This was done to ensure that the chosen literacy level was appropriate, the exercises were achievable, and the instructions were clear enough to enable proper execution while supporting a progressive increase in execution difficulty throughout the program. Additionally, the instructions were validated by first-year medical students as well as individuals without any background in the health sector to ensure that the execution and progression of the exercises were easy to understand for a broader audience. The final version of the PACE-ARSACS program has three primary domains: sitting balance (16 difficulty levels with 1 to 3 exercises per level), standing balance (26 difficulty levels including 3 options for positioning of the feet with 1 to 2 exercises per level), and sit-to-stand transfer (12 difficulty levels with 1 exercise per level).

A standardized assessment tool was developed according to the Streiner and Norman’s model to determine each participant’s individualized initial exercise difficulty level [18]. First, a literature review relating to the impairments and activity limitations found in ARSACS and related outcome measures to quantify them was conducted. Subsequently, the main criteria for the development of the assessment tool were established and included: (1) Evaluation items related to the main impairments and activity limitations associated with the mobility; (2) Applicability to all mobility levels; (3) Discriminative (no floor and/or ceiling effect); (4) Ordinal rating scale that includes the same number of levels for each item (highest score = best performance); (5) Feasibility at home (safe, minimal equipment, environmental considerations); and (6) Maximum evaluation duration of 20–30 min. The selection of items was based on our previous natural history studies [9, 12] and expert consultation. To assess the frequency repartition, discriminative and convergent validity of the items included in the tool, a validation phase was done using the data from a previous natural history study conducted by our research team [9]. The evaluation items in the final version were divided into three components (sit-to-stand transfer, standing balance, and reaching [shift of gravity center]). Each component included two evaluation items and a sub-score of /6. Finally, a decision tree was built to allow the PT to start the program at an appropriate level of difficulty for each domain, considering the three sub-scores. For safety reasons and to allow the participant to become familiar with the program, the initial difficulty level determined by the decision tree was set one level lower than the one corresponding to the assessed capacity.

### Intervention

For all participants, the individualized tailored home-based training program was performed three times a week for a period of 12 weeks. Before starting the training program, a home visit was carried out by a treating PT to teach the PACE program (exercises execution and progression criteria). They also gave them the necessary training equipment and a hard copy of the PACE-ARSACS program. The treating PT supervised the training by conducting follow-up phone call after one week, then every two weeks to ensure safety, assess the participant’s feeling of competence toward the exercises, and monitor the progression of the difficulty level of the program. The participants could also contact the treating PT by phone at any time if they had questions, difficulties, or worries.

### Data Collection

Participants were evaluated during a single session at each of three distinct time points by a trained assessor PT at the Saguenay neuromuscular clinic. The first measurement time (T0) was the control phase and occurred 12 weeks before the start of the training program. The second measurement time (T1) occurred within the two weeks preceding the start of the training program. The final measurement time (T2) occurred within the two weeks following the end of the training program. The assessor PT read the information and consent form to the participants, answered their questions during the T0 assessment, and the participant then signed the form. Outcome measures were administered using standard operating procedures. The administration order of outcomes measures remained constant for all participants, with the order of assessments selected a priori to reduce fatigue during the assessment period. Participant continued to receive their normal care and treatments during the control phase (between T0 and T1). At T1, the developed assessment tool and the decision tree were used for each participant to determine their initial difficulty level.

After the training program (T2), a focus group was conducted by a research professional with expertise in qualitative methods to document the program’s acceptability. Participants were contacted by phone to assess their interest in participating in the group. The sample aimed for optimal variability, including individuals who, during the quantitative evaluation, reported perceiving effects or not perceiving effects, as well as individuals who completed the program, those who dropped out, and individuals at different stages of mobility. The discussion guide was based on the theoretical framework of Sekhon et al. [20], which includes seven constructs (affective attitude, burden, ethicality, intervention coherence, opportunity cost, perceived effectiveness, and self-efficacy). Participants were also asked about barriers and facilitators for program adherence.

### Outcome Measures

A sociodemographic questionnaire was completed at the beginning (T0) to document information about age, sex, height, weight, and mobility levels. Due to feasibility issues, most non-walker participants were not weighed. The genotype was retrieved from the medical record. Self-reported attendance rate was assessed based on the data recorded in the participants’ logbook. The treating physiotherapist completed a logbook after each follow-up call, which included information about the recommended program difficulty level. The participants also completed a logbook after each training session (exercises performed, perceived difficulty, and progression). The self-reported attendance rate was determined by dividing the reported number of exercise sessions completed by the participants by the total number of sessions they were supposed to complete during the study (36 sessions).

All the following tests were performed at each measurement time. *For all participants.* The Berg Balance Scale (BBS) was used to measure balance and risk of falling [21]. It includes 14 items for a maximum score of 56, where a higher score indicates better balance. A score < 45 indicates greater risk of falling. The BBS demonstrated validity in the ARSACS population [22]. The Ottawa Sitting Scale (OSS) was used to assess sitting balance and trunk control [23]. It included 12 items for a maximum score of 48, a higher score reflecting a better sitting balance. The 30-second Chair Stand (30s-CST) was used to assess the sit-to-stand transfer [24]. It measures the number of sit-to-stand transfers performed in 30 s. This test was performed twice, and the average was taken. The Assessment of Life Habits questionnaire (LIFE-H, version 4.0) was used to measure the level of participation in social and daily activities [25, 26]. Each category is on a scale of 10, and a higher score represents a higher participation level. The Scale for the Assessment and Rating of Ataxia (SARA) was used to quantify ataxia severity [27]. The total score is 40, and a higher score represents a more severe ataxia. The validity of SARA was documented in ARSACS, but its reliability remains unknown in this population [28]. Participants were also asked to record any falls that occurred during the training program. *For walker participants only*. The simplified Activities-specific Balance Confidence scale (ABC-S) was used to assess the confidence in standing balance [29, 30]. It includes 15 items, each rated on a 4-point Likert scale (0 = not at all confident; 1 = slightly confident; 2 = moderately confident; 3 = very confident), and the sum of all items (maximum of 45) was calculated and used for analysis. Since all items were answered by all participants, the score was not transformed into percentage. Walking speed was evaluated with the 10-meter Walk Test (10mWT) at both self-selected (s-s) and maximal (max) speed and reported in meters/second (m/s). Validity of the 10mWT was demonstrated in ARSACS and it shown excellent interrater reliability (ICC = 0.960–0.997) [22]. Finally, the time to ascent and descent (separately) 10 steps was recorded in seconds, at both s-s and max speed [31].

### Statistical Analyses

Descriptive statistics (median and range for continuous variables, frequency, and percentage for categorical variables) were used to present participant characteristics and performance in all outcome measures. Participants with < 70% of attendance were excluded from quantitative analyses. Participants were categorized according to their mobility level (walker and non-walker), and characteristics were compared between the two groups using the Mann-Whitney U Test or Chi-Square Test.

Participant performance for each test was compared between all phases (T1 vs. T0 and T2 vs. T1) using the Wilcoxon Signed Rank Test. The individual differences between T1 and T2 performance were calculated for each outcome measure and analyzed using the standard error of measurement (SEM), while the median difference of the whole cohort was computed for both the control (T1-T0) and intervention (T2-T1) phases and compared to the standard error of measurement (SEM). The SEM was computed using the standard deviation (SD) at T0 of the present study and the intraclass correlation coefficient (ICC) found in the literature for ARSACS or a comparable population using the formula $\:{SD}_{T0}\sqrt{1-ICC}$, and the following SEM were used: BERG (1.7) [32], OSS (2.6) [23], ABC-S (1.8) [33], 10mWT s-s (0.0244) [22], 10mWT max (0.0404) [22], 30s-CST (0.9) [34], 10-steps ascent s-s (2.1) [31], 10-steps descent s-s (2.9) [31], 10-steps ascent max (2.0) [31], 10-steps descent max (2.6) [31], and SARA (1.2) [27]. The minimal clinically important difference (MCID) of 0.5 was used for the LIFE-H [35]. A pairwise deletion approach was used to handle missing data due to the small sample size. For all statistical analyses, the significance threshold was set at *p* < 0.1 as it helps exploratory studies to identify clinically meaningful effects [36–38]. Due to the statistical power limitations associated with the number of participants in the study, all p-values should be interpreted with caution, as they are intended to highlight potential signals or trends rather than definitive evidence of statistically significant differences. Data were analyzed using IBM SPSS Statistics for Windows, Version 28.0 (Armonk, NY: IBM Corp).

### Qualitative Analyses

The focus group was audio recorded and the audiotapes were reviewed to conduct a content analysis [39]. The Sekhon et al. framework [20] was used to determine categories and extract data. It is a framework widely used in acceptability studies and can enhance the transferability of the results. Content analysis was performed by a trained qualitative researcher and reviewed by the principal investigator. Content analysis was chosen because the objective was to describe the factors that hinder or promote engagement in the program without interpretation.

## Results

A total of 22 participants were recruited. Seven participants dropped out (32%; three walkers and four non-walkers; three men and four women), and the reasons for leaving were: change in family responsibilities (*n* = 1), health issue (*n* = 2), health issue of a family member (*n* = 1), lack of time (*n* = 1) and disinterest (*n* = 2). One participant (walker) was excluded from the analyses due to self-reported attendance rate below 70% (See the flowchart provided in the Appendix 1). In addition to mobility level (walker or non-walker), the eight participants who did not complete the training program had a similar median age to those who completed the program (median age = 37.5 years; *p* = 0.123). The remaining 14 participants were included in the analysis (see Table 1 for characteristics) and reported a median self-reported attendance rate of 97%. Among them, eight were walkers, six were non-walkers, and 50% were men in the whole group and each subgroup. All participants were considered at risk of falling at T0 (score BBS ≤ 45: see Table 2). No falls due to the training program were reported.

**Table 1:** Participant characteristics

Characteristics	Total (*n* = 14)	Walkers (*n* = 8)	Non-Walkers (*n* = 6)	*p*-value
**Age**, y				
median	42.0	42.5	41.5	0.950
[min – max]	[32–47]	[32–47]	[40–47]	
**Height**, cm	*n* = 10		*n* = 2	
median	163.5	164.5	152.5	0.044
[min – max]	[152.0–184.0]	[153.0–184.0]	[152.0–153.0]	
**Weight**, kg	*n* = 9		*n* = 1	
median	64.0	61.7	89.5	N/A
[min – max]	[45.0–105.0]	[45.0–105.0]	N/A	
**BMI** (kg/m^2^)	*n* = 8			
median	22.9	22.9	N/A	N/A
[min – max]	[19.2–31.0]	[19.2–31.0]		
**Sex**, n (%)				
Men	7 (50.0)	4 (50.0)	3 (50.0)	1.0
Women	7 (50.0)	4 (50.0)	3 (50.0)	
**Genotype**, n (%)				
8844delT / 8844delT	13 (92.9)	7 (87.5)	6 (100)	0.571
8844delT / 4744G > A	1 (7.1)	1 (12.5)	0	
**Indoor mobility**				
Without walking aid	1 (7.1)	1 (12.5)	0	< 0.001
With cane or walker	7 (50.0)	7 (87.5)	0	
Wheelchair	6 (42.9)	0	6 (100)	
**Self-reported attendance rate, %**				
median	97.2	88.9	98.6	0.713
[min – max]	[83–100]	[75–100]	[92–100]	

Results are presented as median. Abbreviations: max: maximum; min: minimum; y: years

### Control Phase

No significant change occurred in all outcomes during the control phase (Table 2). Only two outcomes, the ABC-S scale and the LIFE-H social activities subscale, varied beyond the SEM for the whole cohort. It is worth noting that only walker participants (*n* = 8) performed the ABC-S, 10mWT, and 10-step tests, and none of the non-walker participants could perform a repetition at the 30s-CST.

**Table 2:** Balance, mobility, participation and ataxia severity of participants pre- and post-control phase

Outcome measures (*n*)		Measurement points		Δ (T1-T0)	*p*-value
		T0	T1		
**Balance**	BBS ( /56) (*n* = 14) [min-max]	9.0 [3.0–42.0]	9.0 [2.0–39.0]	0.5	0.719
	OSS (/48) (*n* = 14) [min-max]	35.5 [2.0–46.0]	35.0 [2.0–47.0]	0.5	0.944
	ABC-S ( /45) (*n* = 8) [min-max]	26.0 [20.0–32.0]	29.5 [19.0–35.0]	2.0*	0.600
**Mobility**	10mWT s-s (m/s) (*n* = 8) [min-max]	0.665 [0.240–1.00]	0.655 [0.280–1.01]	0.008	0.779
	10mWT max (m/s) (*n* = 8) [min-max]	0.780 [0.250–1.19]	0.729 [0.390–1.29]	0.016	0.484
	Δ 10mWT (m/s) (*n* = 8) [min-max]	0.119 [0.010–0.400]	0.142 [0.060–0.370]	-0.037	0.208
	30s-CST (n rep) (*n* = 14) [min-max]	0 [0–7.5]	0 [0–8.0]	0	0.854
	10-steps Ascent s-s (s) (*n* = 8) [min-max]	17.8 [9.1–32.7]	15.1 [8.1–25.3]	-1.9	0.161
	10-steps Descent s-s (s) (*n* = 8) [min-max]	21.5 [10.4–45.4]	25.5 [8.0–37.4]	-0.05	0.674
	10-steps Ascent max (s) (*n* = 8) [min-max]	13.1 [6.9–28.7]	11.7 [6.6–23.7]	-0.6	0.327
	10-steps Descent max (s) (*n* = 8) [min-max]	13.7 [8.6–40.3]	18.7 [8.4–33.0]	0.7	0.779
**Participation**	LIFE-H (*n* = 14)				
	Mobility ( /10) [min-max]	6.78 [4.2–8.2]	7.35 [4.2–8.7]	0.26	0.331
	Daily activities ( /10) [min-max]	7.89 [5.5–9.1]	7.88 [5.3–9.0]	-0.01	0.730
	Social activities ( /10) [min-max]	8.71 [6.2–9.7]	8.70 [6.8–9.9]	0.60*	0.551
	Total score ( /10) [min-max]	8.05 [6.1–9.0]	8.17 [5.7–9.1]	0.17	0.551
**Ataxia severity**	SARA ( /40) (*n* = 14) [min-max]	20.5 [9.0–34.0]	19.5 [9.0–34.0]	0.25	0.474

Results are presented as median

* Difference beyond the Standard Error of Measurement (SEM). SEM: BBS = 1.7; OSS = 2.6; ABC-S = 1.8; 10mWT s-s = 0.0244; 10mWT max = 0.0404; 30-s CST = 0.9; 10-step Ascent s-s = 2.1; 10-step Descent s-s = 2.9; 10-step Ascent max = 2.0; 10-step Descent max = 2.6; LIFE-H (MCID) = 0.5; SARA = 1.2

T0: 12 weeks before the training starts, T1:two weeks before the training starts; BBS: Berg Balance Scale; OSS: Ottawa Sitting Scale; ABC-S: Simplified Activities-Specific Balance Confidence scale; 10mWT: 10-meter Walk Test; max: maximum speed; s-s: Self-Selected speed; 30s-CST: 30-second Chair Stand Test; LIFE-H: Assessment of Life Habits questionnaire; max: maximum; MCID: minimal clinically important difference; min: minimum; SARA: Scale for the Assessment and Rating of Ataxia

### Intervention Phase

The effects of the training program on the whole cohort are presented in Table 3. Although the p-value should be interpreted with caution considering the limited number of participants (as stated in the Methods section), a statistically significant improvement was observed regarding ataxia severity. A significant increase was also observed in the daily activities and social activities sub-scores, and the total score of the LIFE-H. A significant decrease in the maximum walking speed was observed, but not outside the SEM.

Only the LIFE-H total score and the ataxia severity (SARA) have improved outside of SEM for the whole cohort. The self-selected walking speed has deteriorated outside the SEM.

**Table 3:** Balance, mobility, participation and ataxia severity of participants pre- and post- intervention phase

Outcome measures (*n*)		Measurement points		ΔT2-T1 (95%CI)	*p*-value
		T1	T2		
**Balance**	BBS ( /56) (*n* = 14) [min-max]	9.0 [2.0–39.0]	11.5 [5.0–42.0]	1.5 (0–3)	0.107
	OSS (/48) (*n* = 14) [min-max]	35.0 [2.0–47.0]	38.0 [6.0–46.0]	1.5 (-1–4)	0.293
	ABC-S ( /45) (*n* = 8) [min-max]	29.5 [19.0–35.0]	28.0 [20–36.0]	0 (-7–7)	0.888
**Mobility**	10mWT s-s (m/s) (*n* = 8) [min-max]	0.655 [0.280–1.01]	0.640 [0.210–1.02]	-0.025* (-0.057–0.018)	0.327
	10mWT max (m/s) (*n* = 8) [min-max]	0.729 [0.390–1.25]	0.708 [0.350–1.25]	-0.039 (-0.050 – -0.015)	***0.017***
	30s-CST (n rep) (*n* = 14) [min-max]	0 [0–8.0]	0 [0-8.5]	0 (0–0.25)	0.257
	10-steps Ascent s-s (s) (*n* = 8) [min-max]	15.1 [8.1–25.3]	15.7 [8.2–26.9]	0.37 (-1.3–2.0)	0.674
	10-steps Descent s-s (s) (*n* = 8) [min-max]	25.5 [8.0-37.4]	22.0 [10.2-40.84]	-0.5 (-7.6–2.2)	0.575
	10-steps Ascent max (s) (*n* = 8) [min-max]	11.7 [6.6–23.7]	12.1 [6.7–25.5]	1.14 (-0.23–2.1)	0.093
	10-steps Descent max (s) (*n* = 8) [min-max]	18.7 [8.4–33.0]	13.1 [9.6–38.3]	-2.3 (-5.7–1.5)	0.310
**Participation**	LIFE-H (*n* = 14)				
	Mobility ( /10) [min-max]	7.35 [4.2–8.7]	7.33 [2.1–8.1]	-0.18 (-0.58–0.61)	0.975
	Daily activities s-s ( /10) [min-max]	7.88 [5.3-9.0]	8.46 [3.7–9.1]	0.31 (0.01–0.87)	***0.084***
	Social activities s-s ( /10) [min-max]	8.70 [6.8–9.9]	9.32 [8.5–10.0]	0.13 (-0.04–1.7)	***0.056***
	Total score ( /10) [min-max]	8.17 [5.7–9.1]	8.73 [4.9–9.1]	0.50* (0.16–0.72)	***0.022***
**Ataxia severity**	SARA ( /40) (*n* = 14) [min-max]	19.5 [9.0–34.0]	18.25 [11.5–31.0]	-1.5* (-2.0 – -0.75)	***0.021***

Presented results are median

* Difference is greater than the Standard Error of Measurement (SEM). SEM: BBS = 1.7; OSS = 2.6; ABC-S = 1.8; 10mWT s-s = 0.0244; 10mWT max = 0.0404; 30-s CST = 0.9; 10-step Ascent s-s = 2.1; 10-step Descent s-s = 2.9; 10-step Ascent max = 2.0; 10-step Descent max = 2.6; LIFE-H (MCID) = 0.5; SARA = 1.2

T1:two weeks before the training starts; T2:two weeks after the training ends; BBS: Berg Balance scale; CI: Confidence interval; OSS: Ottawa Sitting Scale; ABC-S: Simplified-Activities-Specific Balance Confidence scale; 10mWT: 10-meter Walk Test; max: maximum speed; s-s: Self-Selected speed; 30s-CST: 30-second Chair Stand Test; LIFE-H: Assessment of Life Habits questionnaire; MCID: minimal clinically important difference; SARA: Scale for the Assessment and Rating of Ataxia

The individual effects of the training program are presented in Table 4. All participants except one showed improvement outside the SEM in at least one outcome measure, and the mean number of improved outcomes was four (38%). A mean of three outcomes showed worsening beyond the SEM. Eleven out of 14 (79%) participants showed a higher number of outcomes that improved in comparison to those that worsened following the training program. This percentage reached 100% in the non-walker subgroup, where all participants improved in at least three outcomes out of seven.

For balance outcomes, half of the whole cohort improved their balance as measured by the BBS (four out of eight walkers and three out of six non-walkers) and their balance confidence (four out of eight walkers). In addition, almost half of the whole cohort improved their sitting balance and trunk control as measured by the OSS (one out of eight walkers and five out of six non-walkers). For mobility outcomes, half of the whole cohort (four out of eight walkers) worsened outside the SEM for walking speed at comfortable and maximal speed. Positive effects outside the SEM were observed in four out of eight walker participants for 30-CST and 10-step tests. For participation outcomes, between five to seven participants, mostly non-walkers, showed an increase in the LIFE-H scores (sub-scores and/or total score). For ataxia severity, 57% of the participants, evenly distributed in both walker (*n* = 4) and non-walkers (*n* = 4) subgroups, showed an improvement outside the SEM.

**Table 4:**
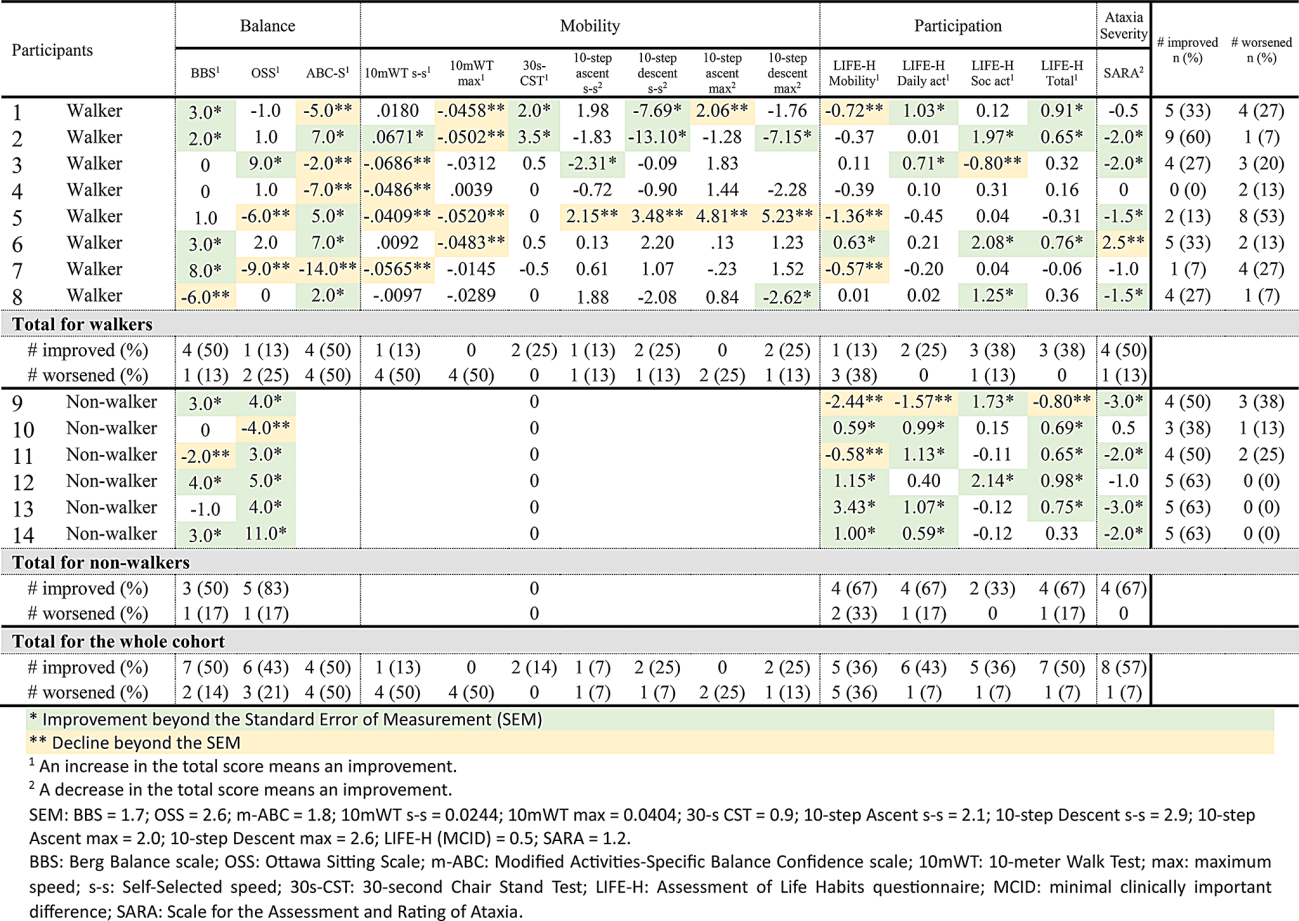
Individual differences between the pre- and post- intervention phase

### Retrospective Acceptability

A total of eight participants took part in the focus group, with a median age of 41.5 years, and 75% (*n* = 6) were women. Among these participants, two were walkers and six were non-walkers. Seven participants completed the intervention program, while one dropped out.

#### Affective attitude

Overall, participants enjoyed the program. Participants reported motivation as a critical factor in the program’s acceptability. The perception of tangible benefits significantly bolstered their motivation to persist into the program, as well as commitment to completing the entire program and regular follow-ups with an expert physiotherapist. As example a participant said: “*I managed to walk with the walker*,* which makes me feel very motivated*,* I’ve seen the effects!*”. Motivation can wane for several reasons. Participants often perceived the exercises as too easy or failed to notice immediate effects, which can demotivate them. Even with initial positive effects, motivation had diminished over time for some participants. The lack of noticeable progression between some exercise levels and the perceived slow pace of improvement contributed to this decline. Seasonal timing, such as conducting the program during a busy summer period, also negatively impacted motivation, as participants find it harder to integrate the program into their routine. The home-based nature of the program presented some challenges including the solitary aspect which can diminish motivation due to the lack of communal energy and support.

#### Burden

Among other barriers that can enhance the perceived burden, participants noted the perceived length of the program. Participants found it difficult to stay engaged with a long-term commitment, especially without significant perceived progress. Integrating the program into daily routines requires significant time management and planning, especially for those with caregiving or working responsibilities. In this sense, participating in the program required specific energy management, especially in the context where energy levels vary from day to day. Regarding this, a participant said: “*I had to do my 20 hours a week for my job*,* so I was really overwhelmed and didn’t have enough energy left to do my workout*”. Also, some exercises caused pain (e.g., side bending hurting the ribs), but were considered beneficial for improving flexibility and daily functional capabilities.

#### Perceived Effectiveness

Even if some participants reported none or little effects, most of them reported various positive effects, such as increased leg strength and muscle tone, improved balance, and increased walking distance. These improvements had valuable effects on the ability to perform activities of daily living. Among them, they noted an improve capacity to use stairs, to be able to stand for longer periods, and to have less difficulty picking up an object from the ground. For example, exercises requiring bending to the side are perceived as helpful in daily life to prevent falls and improve flexibility and balance. Performing the program combined with the advice given by the expert physiotherapist contributed to the development of strategies to avoid falls and perform movements more safely. These benefits extended to enhance confidence and independence in managing their condition. As one participant said: “*It’s always nice to improve one’s physical condition in a degenerative disease*,* because it’s like giving myself the means to have some control over the illness*”.

#### Ethicality

Despite being initially too easy for some, the program was generally well-tailored to participants’ abilities.

#### Intervention coherence

Regular consultations with an expert physiotherapist helped participants understand and adapt exercises to their needs, boosting confidence and correct execution. The physiotherapist’s availability for advice and motivation was highly appreciated. Misunderstandings about exercise difficulty levels and progression can hinder the program’s success. Clear communication from the physiotherapist was essential to ensure participants correctly gauge their progress and adapt exercises appropriately.

#### Opportunity Costs

Some participants had to compromise their usual activities to complete the program according to the issued recommendations. Thus, it could be more difficult to balance the program with their daily tasks (e.g., taking care of their children, doing housework). It could also be challenging to reconcile home exercises with previously conducted gym training.

#### Self-efficacity

Certain external factors may have undermined the participants’ sense of effectiveness. This includes exercises perceived as either too easy, too difficult, or monotonous, as well as not having someone on-site to assist with the exercises. In this regard, the expert physiotherapist’s support is a key element in enhancing the sense of effectiveness. The participants greatly appreciated her availability, social skills, and expertise. Some personal attitudes can also hinder self-efficacy, including inconsistency, procrastination, and lack of structure. Strategies such as consistent effort and effective management of instructional materials can enhance it.

## Discussion

The results of this study, along with those from previous studies [16, 17], suggest that people with ARSACS who follow a training program may experience potential clinical benefits, such as improved balance, increased participation, and reduced ataxia severity. Importantly, this study indicates that those benefits could occur with a tailored training program performed at home under safety constraints. Moreover, the program was generally deemed acceptable by participants.

At baseline, participants demonstrated significant balance impairments and mobility limitations. Only one participant could walk without aid within their homes, while six relied on a wheelchair for indoor mobility. The median maximum walking speed (0.73 m/s) of participants was below the normative walking speed (1.2 m/s) for crossing street safely [40]. This walking speed is lower than that reported in several balance training studies conducted in individuals with degenerative cerebellar disease [41–43]. Moreover, almost all participants were at high risk of falling, with BBS scores below 45 and ABC-S scale scores below 69%, indicating a high likelihood of recurrent falls in a similar population [44]. The median age of the participants was 42.0 years, which is slightly above the beginning of wheelchair use on a daily basis [12]. Unsurprisingly and considering the slowly progressive nature of the disease, the measured variables were stable over the 3-month control period prior the beginning of the training program [9, 10].

The average improvement of 1.5 points on the SARA scale for the whole cohort indicates that participants regained equivalent to one year of the disease progression [9]. This important result is in accordance with previous studies having shown that improvements in ataxia can occur following an intervention in hereditary ataxia [16, 45]. At the cohort level, gains in balance could have been hindered by the high heterogeneity in the performance at balance tests at baseline among participants (BBS: 2.0–39.0 points; OSS:2.0–47 points) combined with the submaximal nature of the program for safety reasons. Although the whole cohort did not reach statistical significance, 50% of the participants showed improvement beyond the SEM for standing balance and balance confidence. These results are consistent with those obtained in two home-based training studies in individuals with ataxia, which did not report significant improvements in balance [46, 47]. One hypothesis for this finding is that individuals may have difficulty sufficiently challenging themselves at home compared to when training is supervised [45, 48]. Otherwise, most of the participants perceived an improvement in sitting balance and trunk control at the end of the training program, particularly in experiencing less difficulty picking up an object from the ground. Those results may stem from the specificity of two out of the three domains of the exercise program: sitting balance and standing balance. However, no significant and clinical changes were observed in the 30-CST (only two walkers out of eight improved outside the SEM) despite that sit-to-stand transfer was the third domain of the exercise program. This is in accordance with the results obtained in our pilot study [16], where it was hypothesized that lack of improvement could be explained by the participant’s caution when executing the transfers. This reason is even more valid in the context of home training. However, improvement outside SEM has been measured in a sub-group of participants confirming the importance to include sit-to-stand transfer exercises in such training program. No positive significant or clinical changes were observed in walking speed. These results align with a systematic review by Barbuto, Kuo, and Stein, which reported that five out of eight balance training studies conducted in individuals with degenerative cerebellar disease did not show significant improvements in walking speed [48]. Notably, some of these studies did not include walking exercises, which may partly explain the lack of improvement in walking speed, as observed in our study [49, 50]. There was a significant improvement in both daily activities and social activities sub-scores of the LIFE-H, as well as the total score. Reasons for this increased participation might be an increase in confidence in their physical abilities and/or a perceived increase in their balance while participating in these activities, as both are factors that have been shown to greatly impact participation [51, 52]. Otherwise, these results reflect the high association between participation level and balance (*r* = 0.71) and ataxia severity (*r* = -0,75) in ARSACS population [15], both being improved by the training program. Taken together, we showed that despite being completed at-home and being sub-maximal for safety reasons, the results obtained with this program are comparable, while being not statistically significant for balance outcomes, with those previously published where participants took part in a supervised 8-week rehabilitation program [16]. Importantly, no less than 18 recessive spastic ataxias described as cerebellum syndrome with motor neuron involvement have been included into a recent classification established by 12 international ataxia experts [53]. By sharing similar mobility-related impairments, this home-training program could be applicable to those populations.

When looking at participant’s individual improvement and decline, we demonstrated that 79% (11 out of 14) of the whole cohort of participants had more tests that improved compared to the ones that declined. This effect is mainly driven by non-walker participants for which the percentage of tests (out of 7) showing improvement outside the SEM individually ranges from 38 to 63% in comparison to 0–38% for the percentage of tests showing decline outside the SEM. This highlights the effectiveness of the training program for non-walking participants. On the other hand, it does not mean that the training is ineffective for walking ARSACS participants. The program was designed with safety as a priority, incorporating a submaximal rehabilitation approach. While exercises that push beyond safety limits might improve balance more significantly, they also increase the risk of falls, which was deliberately avoided [48]. Additionally, non-walking participants may exhibit a distinct profile of deconditioning, as observed in other populations using wheelchairs, which could make them more responsive to the tailored training program [54]. If we consider only the same variables as the non-walking participants (i.e., excluding ABC-S, 10-mWT, 30s-CST and 10-step tests), most walking participants have more tests that have improved than declined. The decrease in mobility outcomes has been previously observed in other interventional studies in ARSACS [16] and myotonic dystrophy type 1 [55] populations. People with ARSACS tend to perform their movements very quickly, which impairs the quality of execution. During the home program instruction, the physiotherapist emphasized the importance of slowing down movement speed to improve the quality of execution of all exercises, promoting effective motor learning [56]. Participants are more aware of their quality of execution after the training, thus underperforming in tests involving speed. Unfortunately, none of the selected outcome measures were able to capture improvements in the quality of movement execution during the tasks, but anecdotally participants reported that the advice of the expert physiotherapist contributed to the development of strategies to avoid falls and perform movements more safely. In future studies, it would be valuable to assess changes in gait using a pressure-sensitive walkway, such as the GAITRite system, to objectively document and validate potential improvements in movement quality and motor control.

Considering the home-based nature of the PACE-ARSACS program with safety as a major endpoint, participants generally found the program acceptable and enjoyed it. They valued the access to an expert treating physiotherapist and reported positive effects on their strength, balance, and ability to perform daily activities. However, challenges such as motivation, exercise progression, and integration into daily routines were noted, indicating areas for potential improvement to enhance overall acceptability and effectiveness. The self-reported attendance rate showed that while home training programs can be a beneficial component of therapeutic options for individuals who are motivated to maintain such a routine, they are not suitable for everyone. Indeed, a significant number of participants dropped out (*n* = 7; 32%). Interestingly, only 2 dropouts were due to disinterest, while most dropouts (*n* = 5) were attributed to major changes in participant’s lives, making it difficult for them to participate in the program actively and autonomously. Those who continue with the program were very committed. When considering mobility limitation of people with ARSACS, their integration into a rehabilitation program is a crucial need. Those programs must be accessible considering that lack of access to health facilities, even in healthy populations, is a barrier to adherence to exercise. Accessibility is then an element that should be taken into consideration when prescribing exercises to ARSACS population and home-training can thus constitute an excellent alternative.

### Limitations

The main limitation of this study is the small sample size that limits the statistical power of the analyses. For this reason, the p-values must be interpreted with caution since there is a risk for type I (false positives) and type II (false negatives) errors. Comparison with the SEM within the individual analysis were made to limit the impact of lack of power. Also, the limited number of participants did not allow for analysis based on sex, which would be of great interest. However, this sample size is bigger than previous [16, 17]. A larger study including more participants would thus be needed to statistically confirm the results. Nevertheless, all the methods used are well supported by the literature and their variability are well known. Sample size is a challenge in rare diseases, and this study, although limited in its statistical power, brings relevant information regarding the potential benefits of home training for the ARSACS population.

## Conclusion

Overall, the developed home-based training program had positive effects on several key measurements, such as participation and ataxia severity. Although the program did not show significant effects on balance and mobility for the entire cohort, individual analysis revealed that at least 50% of participants showed improvements beyond the SEM in standing balance, ataxia severity, and participation, while less than 15% of them showed deterioration. This shows that previous training programs with positive effects that took place in dedicated facilities can be adapted to a home training program with substantial benefits. It is also the first time that the effects of an at-home training program were studied for both walker and non-walker adults with ARSACS. This is even more important since it allows those people to improve their participation and ataxia severity without having to struggle to get to training facilities. Furthermore, it increases the range of potential interventions by making it more accessible to people with ARSACS and other recessive spastic ataxias worldwide. Further studies with larger sample size would be needed to support these findings and allow for sex and mobility (walker vs. non-walker) comparisons.

## Appendix


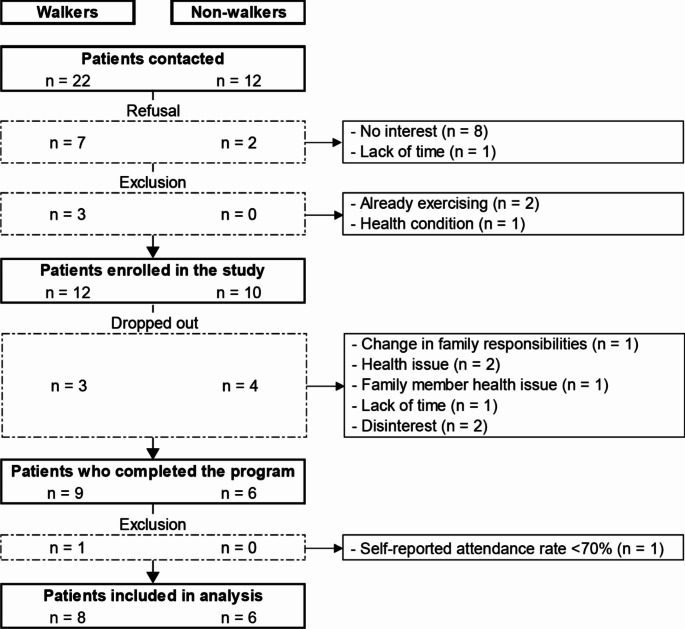


## Data Availability

The datasets used and/or analyzed during the current study are available from the corresponding author on reasonable request following the proper evaluation of the research protocol by the Ethics Review Board of the Centre intégré universitaire de santé et de services sociaux du Saguenay–Lac-St-Jean (Saguenay, Québec, Canada).

## References

[1] Vermeer S, Van de Warrenburg BP, Kamsteeg E-J. ARSACS. In: Adam MP, Ardinger HH, Pagon RA and, et al. editors. GeneReviews^®^ [Internet]. Seattle (WA). University of Washington, Seattle; 2003. p. 8. 20301432

[2] De Braekeleer M, Giasson F, Mathieu J, Roy M, Bouchard JP, Morgan K. Genetic epidemiology of autosomal recessive spastic ataxia of Charlevoix-Saguenay in Northeastern Quebec. Genet Epidemiol. 1993;10:17–25. 10.1002/gepi.1370100103. 8472930 10.1002/gepi.1370100103

[3] Bouhlal Y, Amouri R, El Euch-Fayeche G, Hentati F. Autosomal recessive spastic ataxia of Charlevoix-Saguenay: an overview. Parkinsonism Relat Disord. 2011;17:418–22. 10.1016/j.parkreldis.2011.03.005. 21450511 10.1016/j.parkreldis.2011.03.005

[4] Pilliod J, Moutton S, Lavie J, Maurat E, Hubert C, Bellance N, Anheim M, Forlani S, Mochel F, N’Guyen K, Thauvin-Robinet C, Verny C, Milea D, Lesca G, Koenig M, Rodriguez D, Houcinat N, Van-Gils J, Durand CM, Guichet A, Barth M, Bonneau D, Convers P, Maillart E, Guyant-Marechal L, Hannequin D, Fromager G, Afenjar A, Chantot-Bastaraud S, Valence S, Charles P, Berquin P, Rooryck C, Bouron J, Brice A, Lacombe D, Rossignol R, Stevanin G, Benard G, Burglen L, Durr A. Goizet C and Coupry I. New practical definitions for the diagnosis of autosomal recessive spastic ataxia of Charlevoix-Saguenay. Ann Neurol 2015: 78:871– 86. 10.1002/ana.24509 10.1002/ana.24509 26288984

[5] Guenther G, Lagunes LLF, Alaniz PZ, Woehrlen MCB, de Montellano DJDO, Zapata CMC, García MÁR, Garay CM, Carrillo-Sánchez K, Olivares MJ, Rivas AM, Torres BEV, Saldaña DG, Latorre EAG, Verson CA. First report of spastic ataxia of Charlevoix-Saguenay cases in Mexico. Novel SACS gene mutations identified. Neurology perspectives. 10.1016/j.neurop.2022.07.002

[6] Engert JC, Berube P, Mercier J, Doré C, Lepage P, Ge B, Bouchard JP, Mathieu J, Melançon SB, Schalling M, Lander ES, Morgan K. Hudson TJ and Richter A. ARSACS, a spastic ataxia common in Northeastern Quebec, is caused by mutations in a new gene encoding an 11.5-kb ORF. Nat Genet. 2000;24:120–5. 10655055 10.1038/72769

[7] Bouchard JP, Richter A, Mathieu J, Brunet D, Hudson TJ. Morgan K and melançon SB. Autosomal recessive spastic ataxia of Charlevoix-Saguenay. Neuromuscul Disord. 1998;8:474–9. 9829277 10.1016/s0960-8966(98)00055-8

[8] Briand MM, Rodrigue X, Lessard I, Mathieu J, Brais B, Côté I, Gagnon C. Expanding the clinical description of autosomal recessive spastic ataxia of Charlevoix-Saguenay. J Neurol Sci. 2019;400:39–41. 10.1016/j.jns.2019.03.008. 30901567 10.1016/j.jns.2019.03.008

[9] Gagnon C, Brais B, Lessard I, Lavoie C, Côté I, St-Gelais R, Mathieu J. An exploratory natural history of Ataxia of Charlevoix-Saguenay: A two-year follow-up. Neurology. 2018;91:e1307–11. 30158165 10.1212/WNL.0000000000006290 PMC6177270

[10] Lessard I, Côté I, St-Gelais R, Hébert LJ, Brais B, Mathieu J, Rodrigue X, Gagnon C. Natural history of autosomal recessive spastic Ataxia of Charlevoix-Saguenay: a 4-Year longitudinal study. Cerebellum. 2023. 10.1007/s12311-023-01558-w. 37101017 10.1007/s12311-023-01558-w

[11] Lessard I, St-Gelais R, Hébert LJ, Côté I, Mathieu J, Brais B, Gagnon C. Functional mobility in walking adult population with ataxia of Charlevoix-Saguenay. Orphanet J Rare Dis. 2021;16:432. 10.1186/s13023-021-02054-2. 34649570 10.1186/s13023-021-02054-2 PMC8515729

[12] Gagnon C, Brais B, Lessard I, Lavoie C, Côté I, Mathieu J. From motor performance to participation: A quantitative descriptive study in adults with autosomal recessive spastic ataxia of Charlevoix-Saguenay. Orphanet J Rare Dis. 2018;13:165. 30231904 10.1186/s13023-018-0898-z PMC6146508

[13] Canada Adlspd. Chutes chez les aînés au Canada: Deuxième rapport. https://www.canada.ca/fr/sante-publique/services/promotion-sante/vieillissement-aines/publications/publications-grand-public/chutes-chez-aines-canada-deuxieme-rapport.html. Accessed January 12 2024.

[14] Vogel AP, Rommel N, Oettinger A, Stoll LH, Kraus EM, Gagnon C, Horger M, Krumm P, Timmann D, Storey E, Schols L, Synofzik M. Coordination and timing deficits in speech and swallowing in autosomal recessive spastic ataxia of Charlevoix-Saguenay (ARSACS). J Neurol. 2018;265:2060–70. 10.1007/s00415-018-8950-4. 29968200 10.1007/s00415-018-8950-4

[15] Muslemani S, Lessard I, Lavoie C, Côté I, Brais B, Mathieu J, Gagnon C. Participation and functional independence in adults with recessive spastic ataxia of Charlevoix-Saguenay. Can J Occup Ther. 2022;89(315–25). 10.1177/00084174221088417. 10.1177/00084174221088417 PMC9511234 35469466

[16] Lessard I, Masterman V, Côté I, Gagnon C, Duchesne E. A rehabilitation program to increase balance and mobility in ataxia of Charlevoix-Saguenay: an exploratory study. PLoS ONE. 2022;17:e0279406. 10.1371/journal.pone.0279406. 36576926 10.1371/journal.pone.0279406 PMC9797069

[17] Audet O, Bui HT, Allisse M, Comtois AS, Leone M. Assessment of the impact of an exercise program on the physical and functional capacity in patients with autosomal recessive spastic ataxia of Charlevoix-Saguenay: An exploratory study. Intractable Rare Dis Res. 2018: 7:164– 71. 10.5582/irdr.2018.01060 10.5582/irdr.2018.01060 PMC6119673 30181935

[18] Streiner DL, Norman GR. Health measurement scales: A practical guide to their development and use. New York: Oxford University Press; 2008.

[19] Slade SC, Dionne CE, Underwood M, Buchbinder R, Beck B, Bennell K, Brosseau L, Costa L, Cramp F, Cup E, Feehan L, Ferreira M, Forbes S, Glasziou P, Habets B, Harris S, Hay-Smith J, Hillier S, Hinman R, Holland A, Hondras M, Kelly G, Kent P, Lauret GJ, Long A, Maher C, Morso L, Osteras N, Peterson T, Quinlivan R, Rees K, Regnaux JP, Rietberg M, Saunders D, Skoetz N, Sogaard K, Takken T, van Tulder M, Voet N, Ward L, White C. Consensus on exercise reporting template (CERT): modified Delphi study. Phys Ther. 2016;96:1514–24. 10.2522/ptj.20150668. 27149962 10.2522/ptj.20150668

[20] Sekhon M, Cartwright M, Francis J J. Acceptability of healthcare interventions: an overview of reviews and development of a theoretical framework. BMC Health Serv Res. 2017;17(88). 10.1186/s12913-017-2031-8. 10.1186/s12913-017-2031-8 PMC5267473 28126032

[21] Berg K, Wood-Dauphinee S, Williams JI, Gayton D. Measuring balance in elderly: preliminary development of an instrument. Physiotherapy Can. 1989;41:304–11.

[22] Lessard I, Brais B, Côté I, Lavoie C, Synofzik M, Mathieu J, Gagnon C. Assessing mobility in autosomal recessive spastic Ataxia of Charlevoix-Saguenay population: validity and reliability of four outcome measures. J Neurol Sci. 2018;390:4–9. 29801904 10.1016/j.jns.2018.03.033

[23] Thornton M, Sveistrup H. Intra- and inter-rater reliability and validity of the Ottawa sitting scale: A new tool to characterise sitting balance in acute care patients. Disabil Rehabil. 2010;32:1568–75. 10.3109/09638280903567893. 20662547 10.3109/09638280903567893

[24] Applebaum EV, Breton D, Feng ZW, Ta AT, Walsh K, Chassé K, Robbins SM. Modified 30-second sit to stand test predicts falls in a cohort of institutionalized older veterans. PLoS ONE. 2017;12:e0176946. 10.1371/journal.pone.0176946. 28464024 10.1371/journal.pone.0176946 PMC5413037

[25] Fougeyrollas P, Noreau L. In: INDCP, editor. Assessment of life habits (LIFE-H 4.0) - User guide for teenagers, adults and seniors. Québec (Canada); 2015.

[26] Desrosiers J, Noreau L, Robichaud L, Fougeyrollas P, Rochette A, Viscogliosi C. Validity of the assessment of life habits (LIFE-H) in older adults. J Rehab Med. 2004;36:177–82. 10.1080/16501970410027485 15370734

[27] Schmitz-Hubsch T, du Montcel ST, Baliko L, Berciano J, Boesch S, Depondt C, Giunti P, Globas C, Infante J, Kang JS, Kremer B, Mariotti C, Melegh B, Pandolfo M, Rakowicz M, Ribai P, Rola R, Schols L, Szymanski S, van de Warrenburg BP, Durr A, Klockgether T, Fancellu R. Scale for the assessment and rating of ataxia: development of a new clinical scale. Neurology. 2006;66:1717–20. 16769946 10.1212/01.wnl.0000219042.60538.92

[28] Bourcier D, Bélanger M, Côté I, Brais B, Synofzik M, Brisson JD, Rodrigue X, Gagnon MM, Mathieu J, Gagnon C. Documenting the psychometric properties of the scale for the assessment and rating of ataxia to advance trial readiness of autosomal recessive spastic Ataxia of Charlevoix-Saguenay. J Neurol Sci. 2020;417:117050. 10.1016/j.jns.2020.117050. 32736199 10.1016/j.jns.2020.117050

[29] Filiatrault J, Gauvin L, Fournier M, Parisien M, Robitaille Y, Laforest S, Corriveau H, Richard L. Evidence of the psychometric qualities of a simplified version of the Activities-specific balance confidence scale for community-dwelling seniors. Arch Phys Med Rehabil. 2007;88:664–72. 10.1016/j.apmr.2007.02.003. 17466738 10.1016/j.apmr.2007.02.003

[30] Powell LE, Myers AM. The Activities-specific balance confidence (ABC) scale. J Gerontol Biol Sci Med Sci. 1995;50a:M28–34. 10.1093/gerona/50a.1.m28 7814786

[31] Almeida GJ, Schroeder CA, Gil AB, Fitzgerald GK, Piva SR. Interrater reliability and validity of the stair ascend/descend test in subjects with total knee arthroplasty. Arch Phys Med Rehabil. 2010;91:932–8. 10.1016/j.apmr.2010.02.003. 20510986 10.1016/j.apmr.2010.02.003 PMC2892160

[32] Downs S, Marquez J, Chiarelli P. The Berg balance scale has high intra- and inter-rater reliability but absolute reliability varies across the scale: a systematic review. J Physiother. 2013;59:93–9. 10.1016/s1836-9553(13)70161-9. 23663794 10.1016/S1836-9553(13)70161-9

[33] Dal Bello-Haas V, Klassen L, Sheppard MS, Metcalfe A. Psychometric properties of activity, self-efficacy, and quality-of-life measures in individuals with Parkinson disease. Physiother Can. 2011;63:47–57. 10.3138/ptc.2009-08. 22210979 10.3138/ptc.2009-08 PMC3024195

[34] Wright AA, Cook CE, Baxter GD, Dockerty JD, Abbott JH. A comparison of 3 methodological approaches to defining major clinically important improvement of 4 performance measures in patients with hip osteoarthritis. J Orthop Sports Phys Ther. 2011;41:319–27. 10.2519/jospt.2011.3515. 21335930 10.2519/jospt.2011.3515

[35] Desrosiers J, Rochette A, Noreau L, Bourbonnais D, Bravo G, Bourget A. Long-term changes in participation after stroke. Top Stroke Rehabil. 2006;13:86–96. 10.1310/tsr1304-86. 17082173 10.1310/tsr1304-86

[36] Bae JH, Ku B, Jeon YJ, Kim H, Kim J, Lee H, Kim JY, Kim JU. Radial pulse and electrocardiography modulation by mild thermal stresses applied to feet: an exploratory study with randomized, crossover design. Chin J Integr Med. 2020;26:299–306. 10.1007/s11655-017-2972-0. 29150789 10.1007/s11655-017-2972-0

[37] Hides JA, Franettovich Smith MM, Mendis MD, Smith NA, Cooper AJ, Treleaven J, Leung F, Gardner AJ, McCrory P, Low Choy NL. A prospective investigation of changes in the sensorimotor system following sports concussion. An exploratory study. Musculoskelet Sci Pract. 2017;29:7–19. 10.1016/j.msksp.2017.02.003. 28259770 10.1016/j.msksp.2017.02.003

[38] Buschert VC, Friese U, Teipel SJ, Schneider P, Merensky W, Rujescu D, Möller HJ, Hampel H, Buerger K. Effects of a newly developed cognitive intervention in amnestic mild cognitive impairment and mild Alzheimer’s disease: a pilot study. J Alzheimers Dis 2011: 25:679– 94. 10.3233/jad-2011-100999 10.3233/JAD-2011-100999 21483095

[39] Paillé P, Mucchielli A. L’analyse qualitative en sciences humaines et sociales. Armand Colin; 2021.

[40] Fritz S, Lusardi M. White paper: walking speed: the sixth vital sign. J Geriatr Phys Ther 2009: 3246–9. 20039582

[41] Barbuto S, Kuo SH, Winterbottom L, Lee S, Stern Y, O’Dell M, Stein J. Home aerobic training for cerebellar degenerative diseases: A randomized controlled trial. Cerebellum. 2023: 22:272– 81. 10.1007/s12311-022-01394-4 10.1007/s12311-022-01394-4 PMC8932090 35303255

[42] Keller JL, Bastian AJ. A home balance exercise program improves walking in people with cerebellar ataxia. Neurorehabil Neural Repair. 2014;28:770–8. 10.1177/1545968314522350. 24526707 10.1177/1545968314522350 PMC4133325

[43] Miyai I, Ito M, Hattori N, Mihara M, Hatakenaka M, Yagura H, Sobue G, Nishizawa M. Cerebellar ataxia rehabilitation trial in degenerative cerebellar diseases. Neurorehabil Neural Repair. 2012;26:515–22. 10.1177/1545968311425918. 22140200 10.1177/1545968311425918

[44] Mak MK, Pang MY. Fear of falling is independently associated with recurrent falls in patients with Parkinson’s disease: a 1-year prospective study. J Neurol. 2009;256:1689–95. 10.1007/s00415-009-5184-5. 19479166 10.1007/s00415-009-5184-5

[45] Milne SC, Corben LA, Georgiou-Karistianis N, Delatycki MB, Yiu EM. Rehabilitation for Individuals With Genetic Degenerative Ataxia: A Systematic Review. Neurorehabil Neural Repair. 2017: 31:609– 22. 10.1177/1545968317712469 10.1177/1545968317712469 28595509

[46] Cabanas-Valdés R, Fernández-Lago H, Peláez-Hervás S, Serra-Rusiñol L, López-de-Celis C, Masbernat-Almenara M. Effect of a home-base core stability exercises in hereditary ataxia. A randomized controlled trial. A pilot randomized controlled trial. Mov Disord Clin Pract. 2024;11(666–75). 10.1002/mdc3.14036. 10.1002/mdc3.14036 PMC11145153 38563436

[47] Ilg W, Brötz D, Burkard S, Giese MA, Schöls L, Synofzik M. Long-term effects of coordinative training in degenerative cerebellar disease. Mov Disord. 2010;25:2239–46. 10.1002/mds.23222. 20737551 10.1002/mds.23222

[48] Barbuto S, Kuo SH, Stein J. Investigating the clinical significance and research discrepancies of balance training in degenerative cerebellar disease: A systematic review. Am J Phys Med Rehabil. 2020: 99:989– 98. 10.1097/phm.0000000000001476 10.1097/PHM.0000000000001476 PMC8260091 32467491

[49] Ilg W, Schatton C, Schicks J, Giese MA, Schöls L, Synofzik M. Video game-based coordinative training improves ataxia in children with degenerative ataxia. Neurology. 2012;79:2056–60. 10.1212/WNL.0b013e3182749e67. 23115212 10.1212/WNL.0b013e3182749e67

[50] Wang RY, Huang FY, Soong BW, Huang SF, Yang YR. A randomized controlled pilot trial of game-based training in individuals with spinocerebellar ataxia type 3. Sci Rep. 2018;8:7816. 10.1038/s41598-018-26109-w. 29777115 10.1038/s41598-018-26109-w PMC5959926

[51] Avelino PR, Nascimento LR, Menezes KKP, Sousa GA, Alvarenga MT, Teixeira-Salmela LF, Magalhães JP, Scianni AA. Walking confidence and perceived locomotion ability explain participation after stroke: A cross-sectional experimental study. Acta Neurol Scand. 2022;146:573–7. 10.1111/ane.13682. 36237130 10.1111/ane.13682

[52] Anaby D, Miller WC, Eng JJ, Jarus T, Noreau L. Can personal and environmental factors explain participation of older adults? Disabil Rehabil. 2009;31:1275–82. 10.1080/09638280802572940. 19340617 10.1080/09638280802572940

[53] Beaudin M, Matilla-Dueñas A, Soong BW, Pedroso JL, Barsottini OG, Mitoma H, Tsuji S, Schmahmann JD, Manto M, Rouleau GA, Klein C, Dupre N. The classification of autosomal recessive cerebellar ataxias: A consensus statement from the society for research on the cerebellum and ataxias task force. Cerebellum. 2019;18:1098–125. 10.1007/s12311-019-01052-2. 31267374 10.1007/s12311-019-01052-2 PMC6867988

[54] de Groot S, van der Scheer JW, Bakkum AJ, Adriaansen JJ, Smit CA, Dijkstra C, Post MW, van der Woude LH. Wheelchair-specific fitness of persons with a long-term spinal cord injury: cross-sectional study on effects of time since injury and physical activity level. Disabil Rehabil. 2016;38:1180–6. 10.3109/09638288.2015.1076072. 26308969 10.3109/09638288.2015.1076072

[55] Lessard I, Gaboury S, Gagnon C, Bouchard K, Chapron K, Lavoie M, Lapointe P, Duchesne E. Effects and Acceptability of an Individualized Home-Based 10-Week Training Program in Adults with Myotonic Dystrophy Type 1. J Neuromuscul Dis 2021: 8:137– 49. 10.3233/jnd-200570 10.3233/JND-200570 33252090

[56] Shmuelof L, Krakauer JW, Mazzoni P. How is a motor skill learned? Change and invariance at the levels of task success and trajectory control. J Neurophysiol. 2012;108:578–94. 10.1152/jn.00856.2011. 22514286 10.1152/jn.00856.2011 PMC3404800

